# A Suggestion

**Published:** 1896-05

**Authors:** L. P. Bethel

**Affiliations:** Kent, Ohio


### A Suggestion.

BY L. P. BETHEL, D.D.S., M. D., KENT, OHIO.

Read before the Mississippi Valley Dental Society, Cincinnati, April, 1896.

The subject of instructing the public in dental matters has been discussed in our societies for years, and yet but little has been done toward enlightening the people.

We might continue to discuss this question until doomsday without greater benefit, unless some active steps are taken.

Many methods of imparting this information have been suggested, viz.: well-written articles in pamphlet form for patients, lectures to school-teachers and pupils, addition of subject matter to physiologies, publishing articles in the newspapers, etc., etc.

No one method, however, as outlined, will reach the number of people desired. It seems to me that the most feasible means is through the press, and I will now suggest a new method of utilizing the newspapers to the greatest advantage.

Let the American Dental Association appoint a committee, one of which shall be made editor. The duties of this committee shall be, first, to correspond and arrange with the leading dailies in the United States, one only in each city, to publish one prepared article in the Sunday edition each week until a whole series is printed. The articles to go to the publishers thoroughly edited and ready for publication, and to be gratuitously given, providing an announcement be made each week in the daily advertising their Sunday issue calling attention to the forthcoming article.

I have assurance that this arrangement can be effected. The Sunday paper is suggested because it has a large circulation and is more generally and thoroughly read than the dailies, although the Saturday or Monday issue could be used if desired.

If this be accomplished, the next duty of the committee shall be to appoint prominent men in the profession to write a series of articles, in popular style, bearing on every phase of the subject in hand. Each person to write only one article, and that to contain not more than 1,000 or 1,500 words. When written, these articles should be sent to the committee to be edited and prepared for the press.

When set in type, proof will be sent to the author for revision. When corrected, enough copies will be printed from the galley to supply the papers secured. Proofs of each article to be sent publishers about one week in advance, and the article to appear simultaneously in the desired edition of all the papers, and this continued each week until the whole series is published.

There are about fifty available papers, and they reach fully 12,000,000 readers.

Then, if desired to further extend this knowledge, dentists in towns can get their home paper to republish the articles, without credit, and call special attention to them.

When set in type, the matter can be paged and stereotyped for pamphlet use, if thought advisable. The pamphlets furnished to dentists at cost, would be very cheap and enable them to supply their patients, and also teachers of the public schools that they may read them to the pupils in the school-room, and thus assist in imparting this knowledge.

The whole expense would not be great. If each State and local society would contribute a few dollars, aside from that given by the American Dental Association, there would be ample means to carry out the project in a thorough manner.

And who can estimate the benefit to millions of people and thousands of dentists.

DISCUSSION.

[This paper is the suggestion of a plan to publish in the daily press, throughout the United States, a series of articles upon dentistry in popular form. The object being the distribution of proper information among the masses of the newspaper-reading public.]—REP.

DR. J. E. CRAVENS: I think the best plan to reach the people is through the physician first. He comes in daily contact with the people, and much confidence is reposed in him.

DR. WRIGHT: I don't think it a very good plan to educate the people upon the subject of dentistry. In Europe I had full sway, my patient knew not what I was doing nor why; he simply submitted to the necessary manipulation and paid the bill. When I returned to America and resumed practice in Cincinnati, I found my patients discussing this, that and everything else relating to dentistry. They were familiar with anatomy, physiology, pathology, therapeutics and treatment, with methods and with filling materials, and when it came to criticising the bill, I heartily wished that so much and intimate knowledge had not been spread abroad.

Dr. J. Taft: I, on the other hand, greatly prefer to deal with the intelligent, educated patient than with the ignorant, that Dr. Wright prefers.

Subject passed.

